# Development and internal validation of a CatBoost–SHAP-guided nomogram for significant liver fibrosis in children with chronic hepatitis B

**DOI:** 10.3389/fmed.2026.1927678

**Published:** 2026-10-05

**Authors:** Yuanzhen Zhang, Tingting Wu, Yunan Chang, Xiaorong Peng, Kai Zhu, Yijing Liu, Tingting Shang, Tao Qin, Jing Zhu, Jiao Tian, Ruiqiu Zhao, Hongmei Xu

**Affiliations:** Department of Infectious Diseases, National Clinical Research Center for Children and Adolescents’ Health and Diseases, Ministry of Education Key Laboratory of Child Development and Disorders, Chongqing Key Laboratory of Child Infection and Immunity, Children's Hospital of Chongqing Medical University, Chongqing, China

**Keywords:** CatBoost, liver fibrosis, non-invasive prediction, pediatric chronic hepatitis B, risk stratification, shapley additive explanations

## Abstract

**Background:**

Significant liver fibrosis can develop in children with chronic hepatitis B (CHB), yet liver biopsy is invasive and unsuitable for repeated assessment. We developed an interpretable, non-invasive prediction model for significant fibrosis in pediatric CHB and evaluated whether baseline risk stratification was associated with subsequent treatment outcomes.

**Methods:**

This retrospective study included 232 children with CHB who underwent liver biopsy, with significant fibrosis defined as Scheuer stage S ≥ 2. Patients were randomly assigned to a development cohort (80%) and a held-out test set (20%). A CatBoost model with Bayesian hyperparameter optimization was used to identify the most informative predictors, and these variables were incorporated into a logistic regression nomogram guided by Shapley additive explanations (SHAP). Model performance was evaluated by discrimination, calibration, decision curve analysis, and DeLong testing. Associations between baseline risk groups and longitudinal outcomes were examined using generalized estimating equations adjusted for age and sex and Kaplan–Meier analyses.

**Results:**

Significant fibrosis was identified in 96 children (41.4%). Liver stiffness measurement, alanine aminotransferase, and platelet count were retained in the final model. The nomogram achieved an area under the receiver operating characteristic curve (AUC) of 0.828 (95% CI, 0.767–0.889) in the development cohort and 0.782 (95% CI, 0.637–0.927) in the held-out test cohort, with performance comparable to that of the CatBoost model (*P* = 0.240). Compared with the low-risk group, high-risk patients showed greater longitudinal reductions in alanine and aspartate aminotransferase, whereas longitudinal changes in liver stiffness measurement and hepatitis B virus DNA were similar between groups. Conversely, low-risk patients achieved virological suppression, HBeAg seroconversion, and biochemical response earlier than those classified as high risk.

**Conclusions:**

An interpretable three-variable prediction model based on CatBoost-guided feature selection accurately identified significant fibrosis in children with CHB while maintaining performance comparable to the machine-learning model. Baseline risk stratification was also associated with subsequent treatment outcomes, although external validation is needed before clinical implementation.

## 1 Background

Chronic hepatitis B (CHB) in children remains a major global health challenge and is one of the most common chronic viral liver diseases in this population (1, 2). Despite widespread vaccination, the burden of pediatric CHB remains substantial, and liver fibrosis often progresses silently during childhood (3). Histological studies have shown that many untreated children already have significant hepatic injury, indicating that clinically relevant liver damage can occur early in the disease course. A recent meta-analysis estimated that nearly 40% of untreated children with CHB have moderate-to-severe inflammation or fibrosis, whereas approximately 2% have already developed cirrhosis, underscoring the importance of early identification (4). In parallel, progress toward the WHO 2030 hepatitis elimination targets has been slower than anticipated, further emphasizing the need for reliable, non-invasive approaches to identify pediatric liver fibrosis at an early stage (5).

Over the past decade, non-invasive approaches for assessing liver fibrosis, including transient elastography, two-dimensional shear-wave elastography, and serum biomarker panels, have become widely accepted alternatives to liver biopsy in adults (6–9). Although these methods have shown good diagnostic performance in adult populations, their application in children remains limited by small study cohorts, insufficient standardization, the modest predictive value of individual biomarkers, and concerns regarding model robustness. In addition, the distinct physiological characteristics and disease progression patterns of pediatric CHB limit the direct application of adult-derived diagnostic thresholds and composite scoring systems, leaving an important unmet need for reliable, pediatric-specific non-invasive assessment tools.

Several studies have linked biochemical and hematological parameters to fibrosis severity in children with CHB and developed multivariable non-invasive tools (10–13). However, these models vary in predictor selection, modelling strategy, and validation, and most have been compared mainly with conventional indices such as APRI and FIB-4. More robust and interpretable pediatric-specific models that integrate complementary clinical information are therefore still needed.

To address these limitations, we developed an interpretable prediction model for significant fibrosis in pediatric CHB by integrating hematological parameters with liver stiffness measurement (LSM). A CatBoost gradient-boosting model with Bayesian hyperparameter optimization was used to identify the most informative predictors, and Shapley additive explanations (SHAP) were applied to quantify their relative contributions and improve model interpretability. The selected predictors were subsequently incorporated into a simplified prediction model for individualized, non-invasive risk assessment of significant fibrosis, with the aim of supporting early clinical decision-making and risk stratification in children with CHB.

## 2 Materials and methods

### 2.1 Study population

Pediatric patients with CHB who underwent liver biopsy at the Children's Hospital of Chongqing Medical University between January 2014 and December 2025 were retrospectively enrolled. Patients were eligible if they were younger than 18 years, had a clinical diagnosis of CHB, and had available liver biopsy results. Exclusion criteria included co-infection with other hepatotropic viruses (e.g., hepatitis C virus), autoimmune liver disease, inherited metabolic liver disorders, or hepatic steatosis; severe infection, hematological disorders, or other conditions that could affect blood cell counts; use of medications affecting hematological parameters; and incomplete clinical records.

### 2.2 Data collection and definitions

Demographic, clinical, and laboratory data were obtained from the hospital electronic medical record system. Variables included age, sex, medical history, complete blood counts, liver function tests (alanine aminotransferase [ALT], aspartate aminotransferase [AST], gamma-glutamyl transferase [GGT], total bilirubin (TBIL), and albumin [Alb]), LSM, and hepatitis B virus (HBV) DNA. The study was approved by the Institutional Ethics Committee (No. 2026059). Data were anonymized before analysis, and the requirement for written informed consent was waived.

LSM was measured by two-dimensional shear-wave elastography (ACUSON Sequoia with a 5C1 convex transducer; Siemens Healthineers, Erlangen, Germany). Examinations were performed after 2–4 h of fasting by experienced sonographers. At least 10 valid measurements were obtained from the right hepatic lobe, and the median value was used for analysis. Measurements with an interquartile range-to-median ratio <30% were considered reliable.

Ultrasound-guided percutaneous liver biopsy was performed before antiviral therapy with an 18-gauge needle. Specimens ≥1.5 cm in length containing at least six portal tracts were considered adequate. Fibrosis was staged according to the Scheuer scoring system, with significant fibrosis defined as S ≥ 2. Two experienced hepatopathologists blinded to the clinical and imaging data independently assessed all biopsy specimens, and discrepancies were resolved by consensus.

Virological suppression was defined as HBV DNA <400 IU/mL, biochemical response as normalization of ALT and AST, and HBeAg seroconversion as HBeAg loss with anti-HBe development.

### 2.3 Model development, interpretation and validation

The cohort was stratified by fibrosis status and randomly divided into a development set (80%) and a held-out test set (20%). The development set was used for model training, hyperparameter optimization, SHAP-based feature selection, logistic regression modeling, and cutoff determination, whereas the test set was reserved for independent evaluation.

CatBoost (version 1.2.10) was implemented in Python as a binary classifier with Logloss as the objective function and AUC as the evaluation metric. Hyperparameters were optimized by Bayesian optimization within stratified 5 × 5 nested cross-validation. Search ranges were 4–10 for tree depth, 0.01–0.30 for learning rate, 1–20 for L2 leaf regularization, and 0–5 for bagging temperature. Bayesian optimization included five initial evaluations followed by 15 sequential iterations. Models were trained for up to 2,000 iterations with early stopping after 100 rounds without improvement. Complete-case analysis was performed without imputation, class weighting, or resampling, and random seeds were fixed. The final model was refitted using the complete development set. Optimized hyperparameters are provided in Supplementary Table S1.

SHAP values were used to quantify predictor importance. The highest-ranked predictors were entered into multivariable logistic regression. Nonlinearity was assessed using restricted cubic splines, whereas multicollinearity was evaluated using variance inflation factors (VIFs) and tolerance values. VIF <10 and tolerance >0.1 indicated no significant multicollinearity. The final model was selected by bidirectional stepwise regression based on the Akaike information criterion and presented as a nomogram.

The optimal cutoff was determined in the development set by maximizing the Youden index and applied directly to the test set. Model performance was evaluated using AUC, sensitivity, specificity, positive and negative predictive values, balanced accuracy, and F1 score. AUCs and 95% confidence intervals were estimated using the DeLong method. Calibration was assessed with 1,000 bootstrap resamples, clinical utility by decision curve analysis, and differences in AUC by paired DeLong tests with Holm adjustment.

### 2.4 Statistical analysis

Statistical analyses were performed using Python (version 3.13.14) and R (version 4.6.1). Continuous variables are presented as mean ± standard deviation or median (interquartile range) and compared using Student's *t*-test or the Mann–Whitney *U* test, as appropriate. Categorical variables are presented as number (percentage) and compared using the *χ*^2^ or Fisher's exact test. Correlations with fibrosis stage were assessed using Spearman's rank correlation.

As an exploratory secondary analysis, the development-set cutoff was applied to the full cohort. Longitudinal changes in ALT, AST, LSM, and HBV DNA were analyzed using generalized estimating equation models (GEE) adjusted for age and sex. Models included risk group, categorical time, and group-by-time interaction, with the low-risk group and baseline visit as references. Kaplan–Meier (KM) analysis was used to evaluate time to virological suppression, HBeAg seroconversion, and biochemical response, with differences assessed by the log-rank test. All tests were two-sided, and *P* < 0.05 was considered statistically significant.

## 3 Results

### 3.1 Baseline characteristics

Among the 302 children with CHB who underwent liver biopsy between January 2014 and December 2025, 232 were included in the final analysis (Figure 1). The study population comprised 146 boys and 86 girls, including 96 patients with significant fibrosis (Scheuer stage ≥ S2) and 136 with non-significant fibrosis (Scheuer stage < S2).

**Figure 1:**
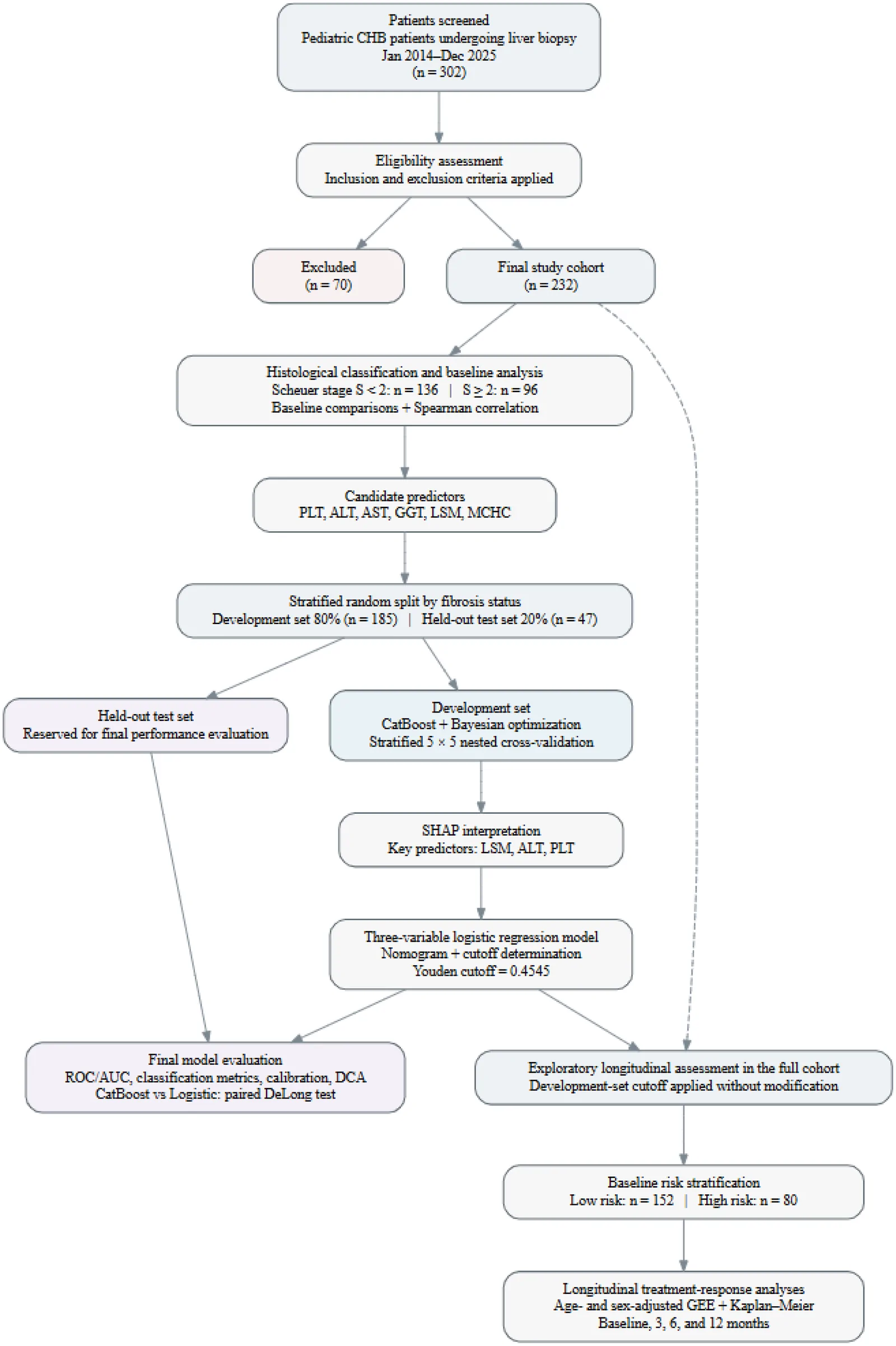
Study design and workflow.

Baseline characteristics are presented in Table 1. Age, sex, HBV DNA levels, white blood cell counts, and hemoglobin concentrations were comparable between the two groups (all *P* > 0.05). Patients with significant fibrosis had lower platelet counts (247.00 ± 59.32 vs. 277.68 ± 69.08 × 10⁹/L; *P* < 0.001) and higher MCHC levels (334.00 [328.00–338.50] vs. 331.00 [324.00–338.00] g/L; *P* = 0.042). ALT, AST, and LSM values were also significantly higher in patients with significant fibrosis (all *P* < 0.001).

**Table 1:** Demographic and clinical characteristics of study participants.

Variable	Significant fibrosis *N* = 96	No significant fibrosis *N* = 136	*P*
Age (year)	7.33 (5.48–11.08)	7.08 (5.15–10.08)	0.4014
Male, *n* (%)	62 (64.58%)	84 (61.76%)	0.7643
HBV DNA (log_10_ IU/mL)	7.80 (6.84–8.24)	7.41 (6.06–8.23)	0.0689
WBC (×10^9^/L)	6.30 (5.33–7.64)	6.58 (5.53–7.75)	0.3942
Hb (g/L)	129.00 (122.75–138.00)	127.00 (121.00–134.25)	0.1539
PLT (×10^9^/L)	247.00 ± 59.32	277.68 ± 69.08	**0**.**0004**
LYM (×10^9^/L)	2.80 (2.20–3.64)	2.92 (2.38–3.65)	0.3334
NEU (×10^9^/L)	2.77 (2.29–3.84)	2.92 (2.32–3.77)	0.6883
MON (×10^9^/L)	0.28 (0.21–0.37)	0.30 (0.22–0.37)	0.4911
MCV(fL)	84.55 (81.62–87.05)	83.95 (81.80–86.43)	0.2907
MCH(pg)	28.30 (27.30–29.33)	27.95 (27.00–29.00)	0.0685
MCHC(g/L)	334.00 (328.00–338.50)	331.00 (324.00–338.00)	**0**.**0422**
HCT(%)	38.80 (36.77–41.12)	38.55 (36.98–40.80)	0.6934
RDW(%)	12.90 (12.47–13.22)	12.80 (12.40–13.20)	0.4451
MPV(fL)	10.40 (9.78–11.60)	10.20 (9.47–11.00)	0.0598
PDW(%)	11.55 (10.05–13.95)	11.30 (10.00–12.80)	0.3997
PCT(%)	0.27 (0.23–0.31)	0.27 (0.24–0.33)	0.3341
Alb(g/L)	44.50 (42.40–46.10)	43.70 (42.63–46.02)	0.408
ALT(U/L)	151.30 (74.50–350.25)	63.00 (40.65–118.05)	**<0**.**001**
AST(U/L)	105.50 (59.75–242.25)	55.00 (41.00–86.20)	**<0**.**001**
GGT(U/L)	19.85 (14.00–42.12)	13.00 (10.00–20.25)	**<0**.**001**
TBIL(*μ*mol/L)	8.00 (6.33–11.05)	8.40 (6.00–11.25)	0.9414
LSM(kPa)	8.30 (6.65–10.12)	6.55 (5.68–7.70)	**<0**.**001**

Continuous variables are presented as mean ± SD or median (IQR), as appropriate; categorical variables are presented as number (percentage).

WBC, white blood cell count; Hb, hemoglobin; PLT, platelet count; NEU, neutrophil count; LYM, lymphocyte count; MON, monocyte count; MCV, mean corpuscular volume; MCH, mean corpuscular hemoglobin; MCHC, mean corpuscular hemoglobin concentration; HCT, hematocrit; RDW, red blood cell distribution width; MPV, mean platelet volume; PDW, platelet distribution width; PCT, plateletcrit; Alb, albumin; ALT, alanine aminotransferase; AST, aspartate aminotransferase; GGT, gamma-glutamyl transferase; TBIL, total bilirubin; LSM, liver stiffness measurement.

Bold values indicate statistical significance at *P* < 0.05.

### 3.2 Correlation analysis between variables and fibrosis stage

Spearman correlation analysis demonstrated that fibrosis stage was positively correlated with ALT (rₛ = 0.395), AST (rₛ = 0.377), LSM (rₛ = 0.357), and GGT (rₛ = 0.290; all *P* < 0.001). Significant but weaker correlations were also observed for HBV DNA (rₛ = 0.150, *P* = 0.023), MCHC (rₛ = 0.139, *P* = 0.035), and MPV (rₛ = 0.132, *P* = 0.045). In contrast, PLT showed a negative correlation with fibrosis stage (rₛ = −0.242, *P* < 0.001). No significant correlations were identified between fibrosis stage and other hematological parameters, albumin, or total bilirubin (all *P* > 0.05; Table 2).

**Table 2:** Spearman's correlation between the variables and the liver fibrosis stage.

Variable	IQR	*r*_s_	*P*
Age (year)	7.25 (5.33–10.35)	0.083	0.2104
HBV DNA (log10 IU/mL)	7.57 (6.58–8.24)	**0**.**15**	**0**.**0227**
WBC (×10^9^/L)	6.43 (5.38–7.70)	−0.068	0.3022
Hb (g/L)	128.00 (121.00–136.00)	0.104	0.1125
PLT (×10^9^/L)	264.00 (218.00–309.25)	−0.242	**0**.**0002**
LYM (×10^9^/L)	2.89 (2.31–3.64)	−0.089	0.1764
NEU (×10^9^/L)	2.88 (2.30–3.82)	−0.034	0.6081
MON (×10^9^/L)	0.30 (0.22–0.37)	−0.01	0.8763
MCV(fL)	84.30 (81.77–86.93)	0.07	0.2893
MCH(pg)	28.05 (27.17–29.10)	0.128	0.0516
MCHC(g/L)	332.00 (325.00–338.00)	**0**.**139**	**0**.**0346**
HCT(%)	38.65 (36.80–40.90)	0.04	0.5448
RDW (%)	12.85 (12.40–13.20)	0.044	0.507
MPV(fL)	10.30 (9.60–11.22)	**0**.**132**	**0**.**0447**
PDW(%)	11.40 (10.00–13.20)	0.063	0.3388
PCT(%)	0.27 (0.23–0.31)	−0.084	0.2007
ALB(g/L)	44.10 (42.40–46.10)	0.053	0.4196
ALT(U/L)	82.50 (47.00–187.15)	**0**.**395**	**<0**.**001**
AST(U/L)	70.10 (44.75–133.05)	**0**.**377**	**<0**.**001**
GGT(U/L)	15.95 (11.00–26.00)	**0**.**290**	**<0**.**001**
TBIL(μmol/L)	8.00 (6.00–11.05)	0.052	0.4346
LSM(kPa)	7.20 (5.90–8.65)	**0**.**357**	**<0**.**001**

Data are presented as Spearman rank correlation coefficients (rₛ) and corresponding *P* values.

WBC, white blood cell count; Hb, Hemoglobin; PLT, platelet count; NEU, neutrophil count; LYM, lymphocyte count; MON, monocyte count; MCV, mean corpuscular volume; MCH, mean corpuscular hemoglobin; MCHC, mean corpuscular hemoglobin concentration; HCT, hematocrit; RDW, red blood cell distribution width; MPV, mean platelet volume; PDW, platelet distribution width; PCT, plateletcrit. Alb, albumin; ALT, alanine aminotransferase; AST, aspartate aminotransferase; LSM, liver stiffness measurement; GGT, gamma-glutamyl transferase; TBIL, total bilirubin.

Bold values indicate statistical significance at *P* < 0.05.

### 3.3 Model development and identification of key predictors

Six candidate variables (PLT, ALT, AST, GGT, LSM, and MCHC) were entered into the CatBoost model. Stratified 5 × 5 nested cross-validation achieved a mean outer-fold AUC of 0.762 ± 0.041. Detailed fold-level validation results are provided in Supplementary Table S2.

SHAP analysis identified LSM, ALT, and PLT as the three most influential predictors (Figures 2A–D). Among all variables, LSM showed the highest mean absolute SHAP value, followed by ALT and PLT. Higher LSM and ALT values contributed positively to model predictions, whereas higher PLT values showed an opposite effect. AST, GGT, and MCHC exhibited relatively limited contributions.

**Figure 2:**
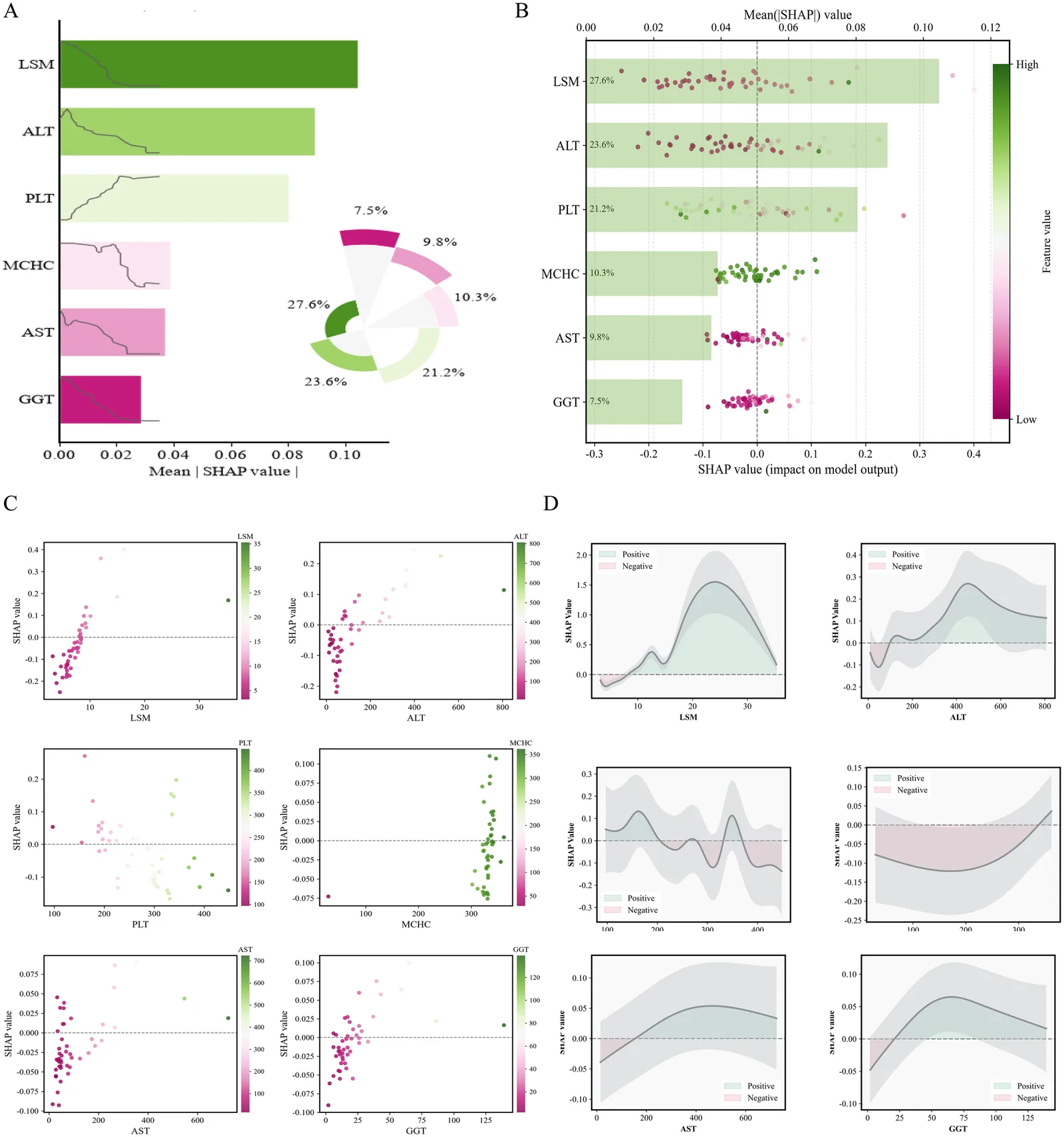
SHAP-based interpretation of the catBoost model for predicting significant fibrosis in children with chronic hepatitis B. **(A)** Feature importance ranked by mean absolute SHAP values of the six candidate predictors, with relative contributions shown in the donut chart. **(B)** SHAP summary plot showing the direction and magnitude of each predictor's contribution to the model output. Each point represents one patient, and color indicates the corresponding feature value. **(C)** SHAP dependence plots showing the relationships between individual predictor values and their SHAP contributions. **(D)** Smoothed SHAP effect curves for the six candidate predictors, with shaded areas representing 95% confidence intervals.

SHAP dependence plots demonstrated that the combination of increased LSM and ALT values with decreased PLT levels was associated with a higher predicted probability of significant fibrosis. Therefore, LSM, ALT, and PLT were selected for development of the simplified logistic regression model.

### 3.4 Performance of the catBoost and logistic regression models

Restricted cubic spline analyses showed no significant evidence of nonlinearity for the associations of PLT, ALT, or LSM with the log odds of significant fibrosis (all *P* for nonlinearity > 0.05; Supplementary Figures 1A–C). No substantial multicollinearity was observed among the predictors, with variance inflation factors ranging from 1.001 to 1.002 and tolerance values ranging from 0.998 to 0.999 (Supplementary Table S3). Because hepatic inflammation may influence LSM, sensitivity analysis using histological inflammation grade showed that LSM remained independently associated with significant fibrosis after adjustment (OR = 1.44, 95% CI 1.24–1.73, *P* < 0.001), with only a minimal change in its coefficient and no significant interaction with inflammation grade (*P* = 0.597; Supplementary Table S4). Therefore, PLT, ALT, and LSM were included as linear terms in the logistic regression model. Bidirectional stepwise selection based on the Akaike information criterion retained all three variables in the final model.

The estimated probability of significant fibrosis was calculated using the following equation: log[P/(1 − P)] = −2.737 − 0.005 × PLT + 0.006 × ALT + 0.370 × LSM

In the development set, the optimal probability threshold determined by the Youden index was 0.4545. At this cutoff, the model achieved a sensitivity of 67.53%, specificity of 87.04%, positive predictive value of 78.79%, negative predictive value of 78.99%, and an AUC of 0.828 (95% CI, 0.767–0.889). When applied to the held-out test set using the prespecified cutoff, the model yielded a sensitivity of 52.63%, specificity of 85.71%, positive predictive value of 71.43%, negative predictive value of 72.73%, and an AUC of 0.782 (95% CI, 0.637–0.927; Supplementary Table S5).

The three-variable model demonstrated the highest AUC compared with individual clinical parameters, conventional non-invasive fibrosis indices, and systemic inflammatory composite indices (Figures 3A,B). Holm-adjusted paired DeLong tests confirmed significant differences between the three-variable model and the comparison models presented in Figure 3C. In the held-out test set, CatBoost achieved an AUC of 0.754, which was not significantly different from that of the logistic regression model (*P* = 0.240).

**Figure 3:**
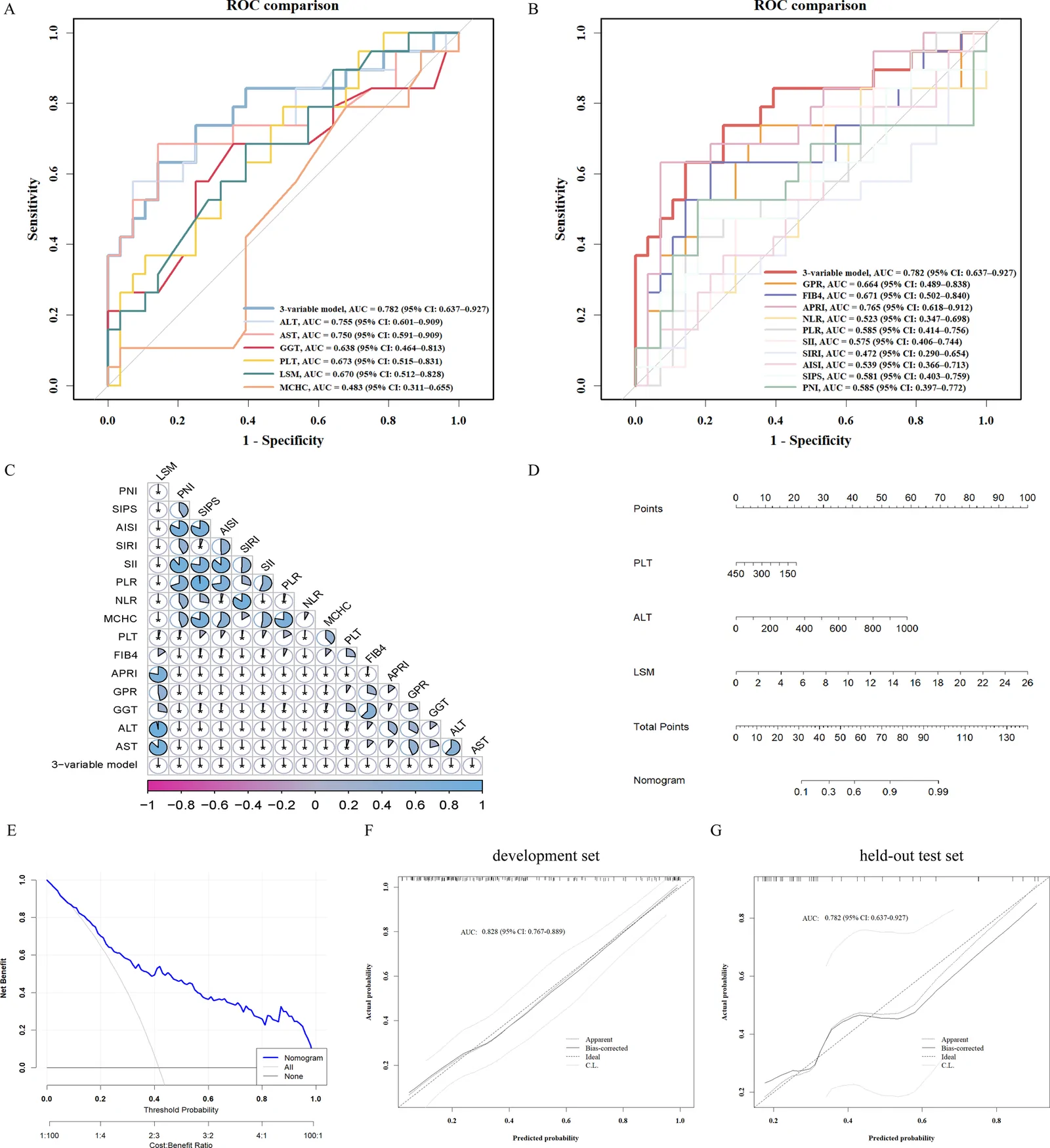
Development and performance evaluation of the three-variable logistic regression model. **(A)** ROC curves comparing the three-variable model with individual clinical indicators in the held-out test set. **(B)** ROC curves comparing the three-variable model with conventional non-invasive fibrosis indices and systemic inflammatory composite indices in the held-out test set. **(C)** Pairwise AUC comparisons using paired DeLong tests with Holm adjustment for multiple comparisons. * indicates Holm-adjusted *P* < 0.05. **(D)** Nomogram incorporating PLT, ALT, and LSM for estimating the probability of significant fibrosis. **(E)** Decision curve analysis showing net clinical benefit across threshold probabilities. **(F)** Calibration curve in the development set. **(G)** Calibration curve in the held-out test set. APRI, aspartate aminotransferase-to-platelet ratio index; FIB-4, fibrosis-4 index; AUC, area under the curve; ROC, receiver operating characteristic curve; PLT, platelet count; ALT, alanine aminotransferase; AST, aspartate aminotransferase; LSM, liver stiffness measurement; MCHC, mean corpuscular hemoglobin concentration; GGT, gamma-glutamyl transferase; GPR, GGT-to-platelet ratio; PNI, prognostic nutritional index; SIPS, systemic inflammatory prognostic score; AISI, aggregate index of systemic inflammation; SIRI, systemic inflammation response index; SII, systemic immune-inflammation index; PLR, platelet-to-lymphocyte ratio; NLR, neutrophil-to-lymphocyte ratio.

The final model was visualized as a nomogram for individualized risk prediction (Figure 3D). Decision curve analysis showed a positive net benefit across the evaluated threshold probability range **(**Figure 3E**)**. Calibration plots demonstrated good agreement between predicted and observed probabilities in the development set, with acceptable calibration maintained in the held-out test set (Figures 3F,G).

### 3.5 Risk stratification and longitudinal assessment

Using the predefined probability cutoff (0.4545), the full cohort was stratified into high-risk (*n* = 80) and low-risk (*n* = 152) groups. At baseline, patients in the high-risk group had higher ALT, AST, and LSM levels than those in the low-risk group, whereas HBV DNA levels were comparable between groups.

Longitudinal changes were analyzed using generalized estimating equation models adjusted for baseline age and sex, with the low-risk group and baseline visit as reference categories. The high-risk group showed significantly higher adjusted baseline ALT, AST, and LSM levels, while no significant baseline difference was observed for HBV DNA. Significant risk-group-by-time interactions were observed for ALT and AST at 3, 6, and 12 months (all *P* < 0.001), with greater decreases from baseline among patients in the high-risk group. No significant interactions between risk group and time were detected for LSM or HBV DNA during follow-up (Table 3).

**Table 3:** Longitudinal analysis of clinical parameters according to risk groups using adjusted GEE models.

Outcome	Time (months)	Low-risk group	High-risk group	Adjusted model					
				Group effect		Time effect		Group × time effect	
				*β* (95% CI)	*P*	β (95% CI)	*P*	β (95% CI)	*P*
ALT	Baseline	103.09 ± 110.05	248.91 ± 152.49	143.69 (106.27 to 181.10)	***<0***.***001***				
	3	78.35 ± 88.54	117.09 ± 110.99			−24.75 (−44.94 to −4.56)	***0***.***016***	−107.07 (−149.00 to −65.15)	***<0***.***001***
	6	46.76 ± 38.31	64.94 ± 68.86			−56.33 (−74.52 to −38.13)	***<0***.***001***	−127.64 (−168.98 to −86.31)	***<0***.***001***
	12	38.98 ± 40.03	44.66 ± 42.66			−64.11 (−83.03 to −45.19)	***<0***.***001***	−140.14 (−180.61 to −99.67)	***<0***.***001***
AST	Baseline	86.71 ± 92.80	188.32 ± 128.47	100.78 (69.16 to 132.41)	***<0***.***001***				
	3	78.12 ± 142.91	92.09 ± 73.13			−8.58 (−35.32 to 18.15)	*0*.*529*	−87.65 (−126.70 to −48.60)	***<0***.***001***
	6	47.34 ± 29.98	57.36 ± 39.69			−39.37 (−54.69 to −24.05)	***<0***.***001***	−91.59 (−125.07 to −58.11)	***<0***.***001***
	12	43.88 ± 35.32	44.41 ± 24.76			−42.83 (−58.97 to −26.69)	***<0***.***001***	−101.08 (−134.17 to −68.00)	***<0***.***001***
LSM	Baseline	6.83 ± 2.26	8.51 ± 2.60	1.61 (0.92 to 2.29)	***<0***.***001***				
	3	7.67 ± 3.14	7.65 ± 2.83			0.63 (−0.67 to 1.93)	*0*.*342*	−1.42 (−3.09 to 0.25)	*0*.*097*
	6	8.05 ± 4.09	8.25 ± 6.04			1.01 (−0.56 to 2.58)	*0*.*208*	−1.19 (−3.87 to 1.48)	*0*.*382*
	12	7.30 ± 1.65	7.74 ± 4.11			0.26 (−0.47 to 0.99)	*0*.*485*	−0.95 (−2.66 to 0.75)	*0*.*274*
HBV DNA	Baseline	7.22 ± 1.53	7.28 ± 1.27	0.08 (−0.29 to 0.45)	*0*.*675*				
	3	4.45 ± 1.56	4.50 ± 1.63			−2.76 (−3.05 to −2.48)	***<0***.***001***	−0.02 (−0.51 to 0.47)	*0*.*935*
	6	3.75 ± 1.43	3.46 ± 1.52			−3.47 (−3.77 to −3.17)	***<0***.***001***	−0.35 (−0.86 to 0.15)	*0*.*171*
	12	3.01 ± 1.15	2.79 ± 1.05			−4.21 (−4.50 to −3.91)	***<0***.***001***	−0.29 (−0.72 to 0.15)	*0*.*195*

The low-risk group and baseline visit were used as the reference categories. The coefficient for risk group represents the between-group difference at baseline; the coefficients for time represent changes from baseline within the low-risk group; and the risk-group-by-time interaction coefficients represent the additional changes over time in the high-risk group relative to the low-risk group.

ALT, alanine aminotransferase; AST, aspartate aminotransferase; LSM, liver stiffness measurement.

Bold values indicate statistical significance at *P* < 0.05.

KM analyses demonstrated significant differences between the two groups in time to virological suppression, HBeAg seroconversion, and biochemical response (*P* = 0.002, *P* < 0.001, and *P* = 0.002, respectively; Figures 4E–G). Compared with the high-risk group, patients classified as low risk achieved these response outcomes earlier.

**Figure 4:**
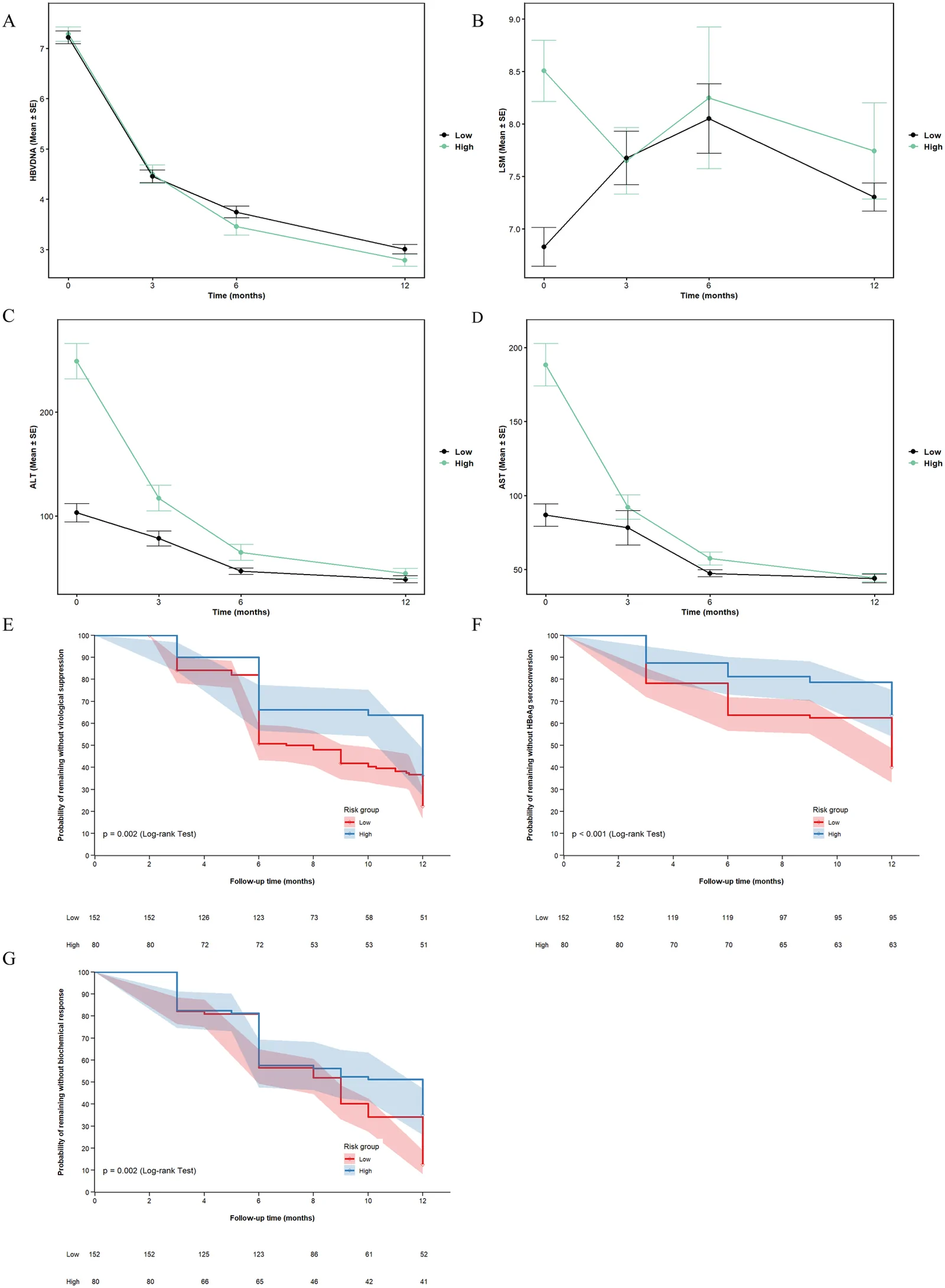
Baseline risk stratification and longitudinal treatment outcomes in children with chronic hepatitis B. **(A–D)** Observed longitudinal changes in HBV DNA, LSM, ALT, and AST at baseline and at 3, 6, and 12 months. Data are presented as mean ± standard error. **(E–G)** Kaplan–Meier analyses of time to virological suppression (HBV DNA <400 IU/mL), HBeAg seroconversion (HBeAg loss with anti-HBe development), and biochemical response (normalization of ALT and AST), respectively. The *y*-axis represents the probability of remaining without the corresponding response; therefore, lower curves indicate earlier or more frequent achievement of the outcome. Between-group differences were assessed using log-rank tests. CHB, chronic hepatitis B; HBV, hepatitis B virus; LSM, liver stiffness measurement; ALT, alanine aminotransferase; AST, aspartate aminotransferase; HBeAg, hepatitis B e antigen; GEE, generalized estimating equation.

## 4 Discussion

In this study, CatBoost and SHAP were used to evaluate the relative importance of six candidate variables, and LSM, ALT, and PLT were identified as the three most informative predictors of significant fibrosis in children with chronic hepatitis B (CHB). These variables were subsequently incorporated into a logistic regression model, which achieved an AUC of 0.828 (95% CI, 0.767–0.889) in the development set and 0.782 (95% CI, 0.637–0.927) in the held-out test set. Its discriminative performance was comparable to that of the CatBoost model. Previous studies in adults with CHB and metabolic dysfunction-associated steatotic liver disease have shown that combining markers related to liver injury and tissue stiffness may improve fibrosis assessment compared with individual markers alone (6, 14). Our findings suggest that a similar multi-marker approach may also be useful in pediatric CHB, although external validation is required.

The selection of LSM, ALT, and PLT is clinically plausible because these variables reflect complementary features of chronic liver disease. LSM is widely used for fibrosis assessment, although hepatic inflammation may transiently increase liver stiffness and thereby overestimate fibrosis severity. Sensitivity analysis showed that LSM remained independently associated with significant fibrosis after adjustment for histological inflammation grade, with minimal change in its regression coefficient and no significant interaction with inflammation grade, suggesting that its predictive contribution was not solely attributable to inflammatory activity. ALT primarily reflects hepatocellular injury, whereas PLT was inversely associated with fibrosis stage. Together, these variables provide complementary predictive information using routinely available laboratory and imaging measurements.

We further examined whether baseline model-derived risk stratification was associated with subsequent longitudinal outcomes. At baseline, patients in the high-risk group had higher ALT, AST and LSM levels and lower platelet counts than those in the low-risk group. During follow-up, significant group-by-time interactions were observed for ALT and AST, with greater absolute declines in the high-risk group. However, because aminotransferase levels were substantially higher at baseline in these patients, the magnitude of the observed reductions was likely influenced, at least in part, by baseline differences. In contrast, longitudinal trajectories of LSM and HBV DNA did not differ significantly between the two risk groups. Kaplan–Meier analyses further showed that patients in the low-risk group achieved virological suppression, HBeAg seroconversion and biochemical response earlier than those in the high-risk group. Collectively, these findings suggest that baseline model-derived risk stratification may be associated with subsequent biochemical and virological outcomes. The clinical and prognostic relevance of these associations warrants further evaluation in prospectively designed longitudinal cohorts.

Inflammation-related hematological indices, including NLR, PLR, SII, SIRI, AISI, SIPS, and PNI, showed lower discriminatory performance than the three-variable model. Age-related variation in blood cell counts and inflammatory responses may partly affect the stability of these indices in pediatric populations. Previous studies have also reported modest or variable predictive performance of individual inflammatory markers in pediatric inflammatory and chronic diseases (15–18). However, this explanation was not directly examined in the present cohort. The current findings therefore indicate only that these indices provided less predictive information than LSM, ALT, and PLT in this population.

Previous pediatric CHB studies have shown that combining routine clinical variables can improve fibrosis assessment compared with single markers or conventional scores such as APRI and FIB-4 (10–13). In the present study, Bayesian-optimized CatBoost and SHAP were used to identify the most informative predictors, which were then reduced to a three-variable logistic nomogram without an apparent loss of discrimination. In addition to conventional fibrosis scores, we also compared the model with a broader panel of inflammation-related composite indices, allowing its performance to be assessed against a wider range of routinely available markers.

This study has several limitations. First, the retrospective single-center design and relatively small held-out test set limit the generalizability and precision of the performance estimates. As the model has undergone only internal validation, the nomogram should be considered investigational and is not yet ready for routine clinical use; external multicenter and prospective validation is required. Second, although liver biopsy served as the reference standard, longitudinal fibrosis progression could not be assessed because repeated biopsies were unavailable. Third, the probability cutoff was derived in the development set and applied to the full cohort for longitudinal analyses, so these findings should be regarded as exploratory rather than independent prognostic validation. Finally, multiple model comparisons and longitudinal outcomes were examined, and some nominally significant findings may reflect chance associations.

## 5 Conclusion

We developed an interpretable machine learning–assisted model integrating PLT, ALT, and LSM for significant fibrosis prediction in children with CHB. The model provided risk stratification and was associated with subsequent treatment-related response patterns. Further external validation and prospective evaluation are needed to determine its clinical applicability.

## Data Availability

The raw data supporting the conclusions of this article will be made available by the authors, without undue reservation.
