# Supplementary material for: Development and internal validation of a CatBoost–SHAP-guided nomogram for significant liver fibrosis in children with chronic hepatitis B
Source: Front Med (Lausanne). 2026 Oct 5;13:1927678. doi: 10.3389/fmed.2026.1927678 (PMC13677468; doi:10.3389/fmed.2026.1927678)
Supplement: Supplementary file 1 [file Supplementaryfile1.docx]

**Supplementary Table S1. Final CatBoost model settings selected in the complete development set**

| **Parameter** | **Final value** |
| --- | --- |
| Tree depth | 5 |
| Learning rate | 0.1298 |
| L2 leaf regularization | 19.5561 |
| Bagging temperature | 0.4445 |
| Number of boosting iterations | 48 |
| Best mean inner cross-validated AUC | 0.8209 |
| Bayesian optimization seed | 2026 |
| Model seed | 42 |

**Note:** Hyperparameters were optimized within the development set using Bayesian optimization with stratified five-fold inner cross-validation. The optimization included five initial random evaluations and 15 sequential iterations. The number of boosting iterations was determined from the median optimal iteration obtained during inner cross-validation. Random seeds were fixed to ensure reproducibility. AUC, area under the receiver operating characteristic curve.

**Supplementary Table S2. Hyperparameter selection and performance across the five outer validation folds of nested cross-validation**

| **Outer fold** | **Inner CV AUC** | **Iterations** | **Depth** | **Learning rate** | **L2 leaf reg.** | **Bagging temperature** | **Outer-fold AUC** | **Accuracy** | **Balanced accuracy** | **Sensitivity** | **Specificity** | **PPV** | **NPV** | **F1 score** |
| --- | --- | --- | --- | --- | --- | --- | --- | --- | --- | --- | --- | --- | --- | --- |
| 1 | 0.818 | 69 | 7 | 0.0876 | 12.6728 | 1.6595 | 0.783 | 0.703 | 0.671 | 0.438 | 0.905 | 0.778 | 0.679 | 0.560 |
| 2 | 0.837 | 19 | 5 | 0.1017 | 3.8047 | 4.6175 | 0.702 | 0.649 | 0.616 | 0.375 | 0.857 | 0.667 | 0.643 | 0.480 |
| 3 | 0.822 | 14 | 7 | 0.0100 | 17.2907 | 0.0000 | 0.752 | 0.730 | 0.688 | 0.467 | 0.909 | 0.778 | 0.714 | 0.583 |
| 4 | 0.823 | 6 | 5 | 0.1968 | 13.6740 | 3.7990 | 0.762 | 0.676 | 0.653 | 0.533 | 0.773 | 0.615 | 0.708 | 0.571 |
| 5 | 0.817 | 70 | 4 | 0.3000 | 15.6387 | 1.8666 | 0.812 | 0.784 | 0.755 | 0.600 | 0.909 | 0.818 | 0.769 | 0.692 |
| Mean ± SD | 0.823 ± 0.008 | / | / | / | / | / | 0.762 ± 0.041 | 0.708 ± 0.052 | 0.677 ± 0.051 | 0.483 ± 0.087 | 0.871 ± 0.059 | 0.731 ± 0.086 | 0.703 ± 0.047 | 0.577 ± 0.076 |

**Note:** Within each outer training fold, CatBoost hyperparameters were optimized using Bayesian optimization with stratified five-fold inner cross-validation. The optimized model was then evaluated in the corresponding outer validation fold, which was not used for hyperparameter selection. AUC was calculated from predicted probabilities and was independent of the classification threshold. Accuracy, balanced accuracy, sensitivity, specificity, positive predictive value, negative predictive value, and F1 score were calculated using a fixed probability threshold of 0.50. AUC, area under the receiver operating characteristic curve; CV, cross-validation; PPV, positive predictive value; NPV, negative predictive value; SD, standard deviation.
