# Supplementary material for: Development and internal validation of a CatBoost–SHAP-guided nomogram for significant liver fibrosis in children with chronic hepatitis B
Source: Front Med (Lausanne). 2026 Oct 5;13:1927678. doi: 10.3389/fmed.2026.1927678 (PMC13677468; doi:10.3389/fmed.2026.1927678)
Supplement: Supplementary file 2 [file Supplementaryfile2.docx]

**Supplementary Table S3. Assessment of multicollinearity among predictors in the logistic regression model**

| **Predictor** | **Variance inflation factor** | **Tolerance** |
| --- | --- | --- |
| PLT | 1.001648 | 0.998355 |
| ALT | 1.001145 | 0.998856 |
| LSM | 1.001280 | 0.998722 |

*Abbreviations: ALT, alanine aminotransferase; LSM, liver stiffness measurement; PLT, platelet count; VIF, variance inflation factor. Tolerance was calculated as 1/VIF.*

**Supplementary Table S4. Sensitivity analysis assessing the potential influence of histological inflammation on the association between LSM and significant fibrosis**

| **Analysis** | **Estimate** | **95% CI** | **P value** |
| --- | --- | --- | --- |
| Histological inflammation grade vs. LSM | Spearman rₛ = 0.159 | — | 0.015 |
| LSM in original model | OR = 1.447 | 1.250–1.722 | <0.001 |
| LSM after adjustment for histological inflammation grade | OR = 1.444 | 1.242–1.727 | <0.001 |
| LSM × inflammation grade interaction | OR = 1.082 | 0.826–1.474 | 0.597 |

**Supplementary Table S5. Predictive performance of the three-variable model in the development and independent test sets**

| **Performance measure** | **Development set** | **Independent test set** |
| --- | --- | --- |
| AUC (95% CI) | 0.828 (0.767–0.889) | 0.7820 (0.637–0.927) |
| Cutoff | 0.4545 | 0.4545 |
| Sensitivity | 0.6753 | 0.5263 |
| Specificity | 0.8704 | 0.8571 |
| PPV | 0.7879 | 0.7143 |
| NPV | 0.7899 | 0.7273 |
| Balanced accuracy | 0.7728 | 0.6917 |
| F1 score | 0.7273 | 0.6061 |
| TP, n | 52 | 10 |
| FN, n | 25 | 9 |
| TN, n | 94 | 24 |
| FP, n | 14 | 4 |

**Note:** The same cutoff (0.4545) derived in the development set was applied to the independent test set. AUC, area under the receiver operating characteristic curve; CI, confidence interval; PPV, positive predictive value; NPV, negative predictive value; TP, true positive; FN, false negative; TN, true negative; FP, false positive.
